# Clinical utility of the FilmArray® meningitis/encephalitis panel in children with suspected central nervous system infection in a low-resource setting – a prospective study in Southwestern Uganda

**DOI:** 10.1186/s12879-025-10732-w

**Published:** 2025-03-22

**Authors:** Reza Rasti, Elias Kumbakumba, Deborah Nanjebe, Phuthumani Mlotshwa, Milly Nassejje, John Mzee, Stephen Businge, Gilbert Akankwasa, Dan Nyehangane, Jesper Gantelius, Yap Boum, Andreas Mårtensson, Juliet Mwanga-Amumpaire, Tobias Alfvén, Giulia Gaudenzi

**Affiliations:** 1 Department of Global Public Health, Karolinska Institutet, Stockholm, Sweden; 2 Department of Paediatrics and Child Health, Faculty of Medicine, Mbarara University of Science and Technology, Mbarara, Uganda; 3 Epicentre Mbarara Research Center, Mbarara, Uganda; 4 Holy Innocents Children’s Hospital, Mbarara, Uganda; 5 Division of Nanobiotechnology, Department of Protein Science, Science for Life Laboratory, KTH Royal Institute of Technology, Stockholm, Sweden; 6 Department of Women’s and Children’s Health, Global Health & Migration Unit, Uppsala University, Uppsala, Sweden; 7 Department of Infectious Diseases, Uppsala University Hospital, Uppsala, Sweden; 8 Sachs’ Children and Youth Hospital, South General Hospital, Stockholm, Sweden

**Keywords:** Molecular diagnostic techniques, Central nervous system infections, Meningitis, Paediatrics, Global health, FilmArray

## Abstract

**Background:**

In low-resource settings, limited laboratory capacity adds to the burden of central nervous system (CNS) infections in children and spurs overuse of antibiotics. The commercially available BioFire® FilmArray® Meningitis/Encephalitis Panel (FA-ME) with its capability to simultaneously detect 14 pathogens in cerebrospinal fluid (CSF), could potentially narrow such a diagnostic gap.

**Methods:**

In Mbarara, Uganda, we compared clinical utility (clinical turnaround time [cTAT], microbial yield, and influence on patient outcome and antibiotic exposure) of FA-ME with bacterial culture, in children 0–12 years with suspected CNS infection.

**Results:**

Of 212 enrolled children, CSF was sampled from 194. All samples underwent bacterial culture, of which 193 also underwent FA-ME analyses. FA-ME analyses prospectively influenced care for 169 of the 193 patients, and they constituted an ‘Index group’. The remaining 43/212 patients constituted a ‘Reference group’. Of all 194 CSF-sampled patients, 87% (168) had received antibiotics before lumbar puncture. Median cTAT for FA-ME was 4.2 h, vs. two days for culture. Bacterial yield was 12% (24/193) and 1.5% (3/194) for FA-ME and culture, respectively. FA-ME viral yield was 12% (23/193). Fatality rate was 14% in the Index group vs. 19% in the Reference group (*P* = 0.20). From clinician receival of FA-ME results, median antibiotic exposure was 6 days for bacteria-negative vs. 13 days for bacteria-positive patients (*P* = 0.03). Median hospitalization duration was 7 vs. 12 days for FA-ME negative and positive patients, respectively (*P* < 0.01).

**Conclusions:**

In this setting, clinical FA-ME utility was found in a higher and faster microbial yield and shortened hospitalization and antibiotic exposure of patients without CSF pathology. More epidemiologically customized pathogen panels may increase FA-ME utility locally, although its use in similar settings would require major cost reductions.

**Trial registration:**

The trial was registered with clinicaltrials.gov (NCT03900091) in March 2019, and its protocol was published in November 2020.

**Supplementary Information:**

The online version contains supplementary material available at 10.1186/s12879-025-10732-w.

## Introduction

Although there is a higher risk of children being affected by central nervous system (CNS) infections than others, their clinical presentation is often more diffuse than it is for adults [1, 2]. Prompt and effective diagnosis and treatment of CNS infections, particularly bacterial meningitis, are crucial for favourable outcomes due to their high morbidity and mortality [2–5].

Annually, 2.9 million cases of bacterial meningitis occur worldwide, with a disproportionally higher incidence in low-income countries [6]. In such contexts, CNS infections account for 2.5–3.5% of deaths among children under the age of five years [7]. Health systems in low-resource settings (LRS) often lack quality-standard or accredited clinical laboratories with the capacity to provide reliable results in time for clinicians to accurately diagnose CNS infections. Discerning CNS aetiology thus becomes a challenging task that impedes appropriate and timely treatment of patients, allows for over-treatment with empirical broad-spectrum drugs, and adds to the burden of disease and the spread of antimicrobial resistance [2, 8–11]. Even where the means are available for bacterial culture – the gold-standard for identification of bacteria in cerebrospinal fluid (CSF) – it is a time-consuming process with a low microbial yield that is further decreased by the frequent empiric administration of antibiotics prior to lumbar puncture (LP) [12]. Consequently, a bacterial culture fails to fulfil the requirements for the swift and accurate diagnostics needed for effective clinical management of bacterial CNS infections [2, 3, 13, 14]. To detect viruses in CSF, advanced techniques such as viral culture or molecular nucleic acid testing using polymerase chain reaction (PCR) are required, yet these are often in shortage in LRS [14]. Hence, there is a need for accurate, rapid, and multiplex diagnostic methods for CSF microbiology with the potential for use in LRS [2, 10, 15].

**Fig. 1:**
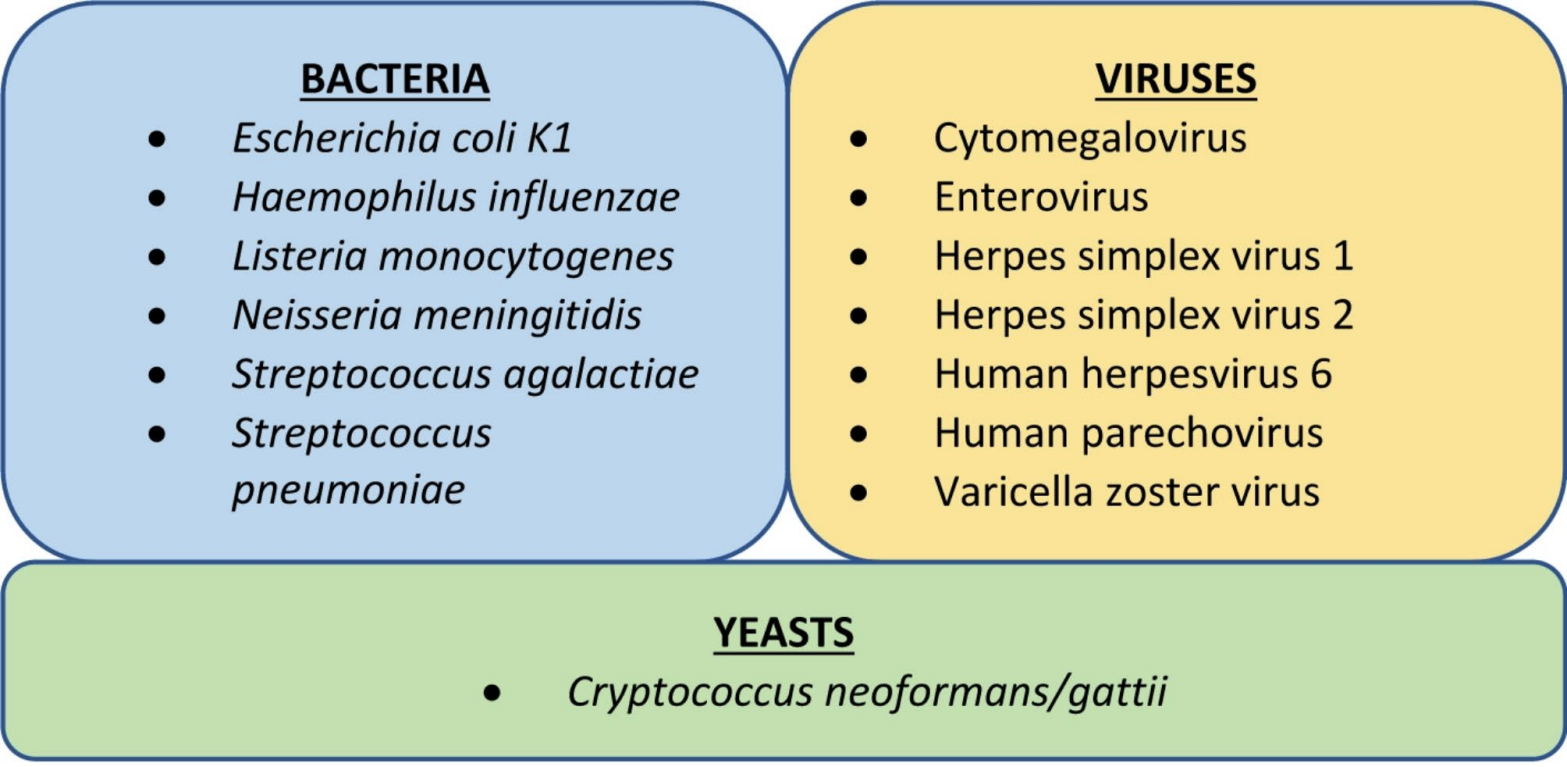
Microorganisms detectable by the FilmArray ME panel [18]

Multiplex molecular methods for rapid identification of a range of pathogens in CSF samples are now increasingly used in CNS infection diagnostics in well-resourced settings [3, 14, 16]. The BioFire® FilmArray® Meningitis/Encephalitis Panel (FA-ME) is the first commercially available multiplex PCR assay, capable of simultaneous detection of 14 pathogens (Fig. 1) in minimal CSF volumes, offering reduced turnaround time and high diagnostic accuracy [1, 3, 17–19]. While its user-friendliness and multiplexity have the potential to narrow the diagnostic gap of CNS infection diagnostics in resource-limited settings, the current cost and logistical needs of FA-ME might deter its implementation [14, 18, 20].

Despite extensive literature on the use and performance of FA-ME, most studies focus on diagnostic accuracy, with only a few conducting prospective field evaluations to assess its clinical utility [21]. Moreover, very few of these studies were conducted in LRS [22, 23]. Therefore, justification for FA-ME usage in LRS is challenging since there are presently few publications on the potential clinical advantages of its use in paediatric CNS infection diagnosis.

The aim of this study was to prospectively evaluate the clinical utility of FA-ME in children with suspected central nervous system infections in a low-resource setting in southwestern Uganda. This was done by comparing its clinical turnaround time and microbial yield, as well as its impact on patient outcome (cure/death) and duration of antibiotics exposure, to those of the diagnostic method (culture) otherwise available in the setting.

## Materials and methods

### Study design and setting

This study was part of the Paediatric Infection Point-of-Care (PI-POC) trial, a prospective observational trial investigating and evaluating novel methods for diagnosing childhood CNS infections in low-resource settings. The trial protocol is published [24] and registered with clinicaltrials.gov (NCT03900091). Between March 2019 and July 2020, clinical data and samples (blood and CSF) were collected from children aged 0–12 years who were hospitalised with suspected CNS infection at the paediatric clinic of Mbarara Regional Referral Hospital (MRRH) and at the Holy Innocents Children’s Hospital (HICH), in Mbarara, southwestern Uganda.

Located in the so-called “Sub-Saharan meningitis belt”, due to its periodic CNS infection outbreaks, Uganda is a low-income country with a population of about 42 million, of which 400,000 reside in its southwestern Mbarara district [25, 26]. In Uganda, 46 out of 1,000 children born alive die before the age of 5 years, with 2.4% of such deaths being attributed to meningitis [27].

MRRH is a public regional referral hospital in southwestern Uganda with a 70-bed paediatric ward. It serves around 5,000 children annually, many of whom are admitted due to infections. The hospital is located on the campus of Mbarara University of Science and Technology and houses the clinical facilities of the university’s medical faculty [24].

HICH is a private non-profit paediatric hospital with 60 beds, an emergency department, and basic clinical laboratory services, providing in- and outpatient care.

Epicentre, affiliated with Médecins Sans Frontières, is a research centre on the Mbarara University of Science and Technology and MRRH campus. It specializes in field epidemiology and clinical research, is equipped with a biosafety level 3 laboratory, and is accredited for Good Clinical Laboratory Practice. Their clinical laboratory capabilities cover mycobacteriology, parasitology, microbiology, molecular biology, serology, biochemistry, haematology, and biobanking [28]. As part of the PI-POC trial objectives, a FA-ME instrument was procured and installed at the Epicentre laboratory in May 2019.

### Study population

Between March 2019 and July 2020, paediatric patients meeting predetermined inclusion criteria for suspected CNS infection at the two hospitals were enrolled (Appendix 1). They underwent a diagnostic work-up, including routine LP and venepuncture. However, due to shifting clinical priorities and a COVID-19 lockdown in the country, there was no new recruitment between March 31 and June 25, 2020. The study started two months before the FA-ME instrument was installed in May 2019, allowing for the retrospective FA-ME analysis of 24 CSF samples collected earlier.

### Logistics and laboratory procedures

LP (one per patient) was performed by the lead medical officer of the PI-POC study, mostly during admission hours seven days a week from 8 AM – 6 PM. Patient samples were collected at the two hospitals and transported to the Epicentre laboratory by the study nurses within 20 min of the LP; from MRRH by foot, from HICH by motorcycle taxi. Geographically, Epicentre is located adjacent to MRRH, with HICH located 2.5 km away. CSF analyses, including culture, FA-ME, and cytology, were conducted at Epicentre mainly during working hours, 8AM – 6 PM.

CSF samples were directly inoculated on culture media before any other testing: Gram staining, cell counting, biochemistry and FA-ME. However, samples grossly contaminated with blood in traumatic lumbar punctures were exempted from cell counting. These samples were centrifuged for five minutes at 3,800 rpm using the Hettich EBA 20 centrifuge (Hettich, Germany) and the sediments resuspended with a vortex before using them to inoculate the culture media and preparation of Gram smears.

For cell counting, 10 μl of uncentrifuged CSF was dispensed into a counting chamber. If more than 10 WBC/μL were observed, differential white cell counts were conducted and the percentage proportions of polymorphonuclear and mononuclear cells reported. A sterile pipette was used to systematically inoculate either the uncentrifuged CSF or the vortexed sediment onto blood, chocolate CHROMagar™ Orientation and MacConkey agar plates. The blood and chocolate agar plates were cultured anaerobically at 35–37 °C while the MacConkey agar and CHROMagar™ Orientation plates were cultured aerobically at the same temperature. Plates were examined at 24 h and, if negative, were re-incubated and re-examined at 48 h. Culture-based identification was performed. When indicated by cytology, Gram smears were prepared on the sediment. CSF samples collected prior to the instalment of the FA-ME instrument underwent standard culture analyses as described above, but an aliquot sample was stored in -80 °C biobank facilities for subsequent analyses. FA-ME analyses were performed according to the manufacturer’s instruction, using 200 μL of CSF. Laboratory results were reported to HICH by email and to MRRH by hand.

### Data management and statistical methods

Pseudonymised study data were entered in Case Report and Laboratory Result Forms on paper by the study investigator. Data officers transferred the data to a digital database hosted by the REDCap electronic data capture tools hosted at the Karolinska Institutet; quality control was performed on the dataset [29, 30]. Descriptive statistical analyses and graphical presentations were done using STATA/BE 17.0 and GraphPad Prism 9.2.0. The Mann Whitney U test was used to analyse for group differences in non-normally distributed continuous variables (e.g. age and clinical turnaround time), and the chi-square test for categorical variables. The statistical significance level was set at *P* < 0.05. For continuous variables, the median and interquartile range (IQR) were calculated.

### Definition of clinical turnaround time (cTAT)

**Fig. 2:**
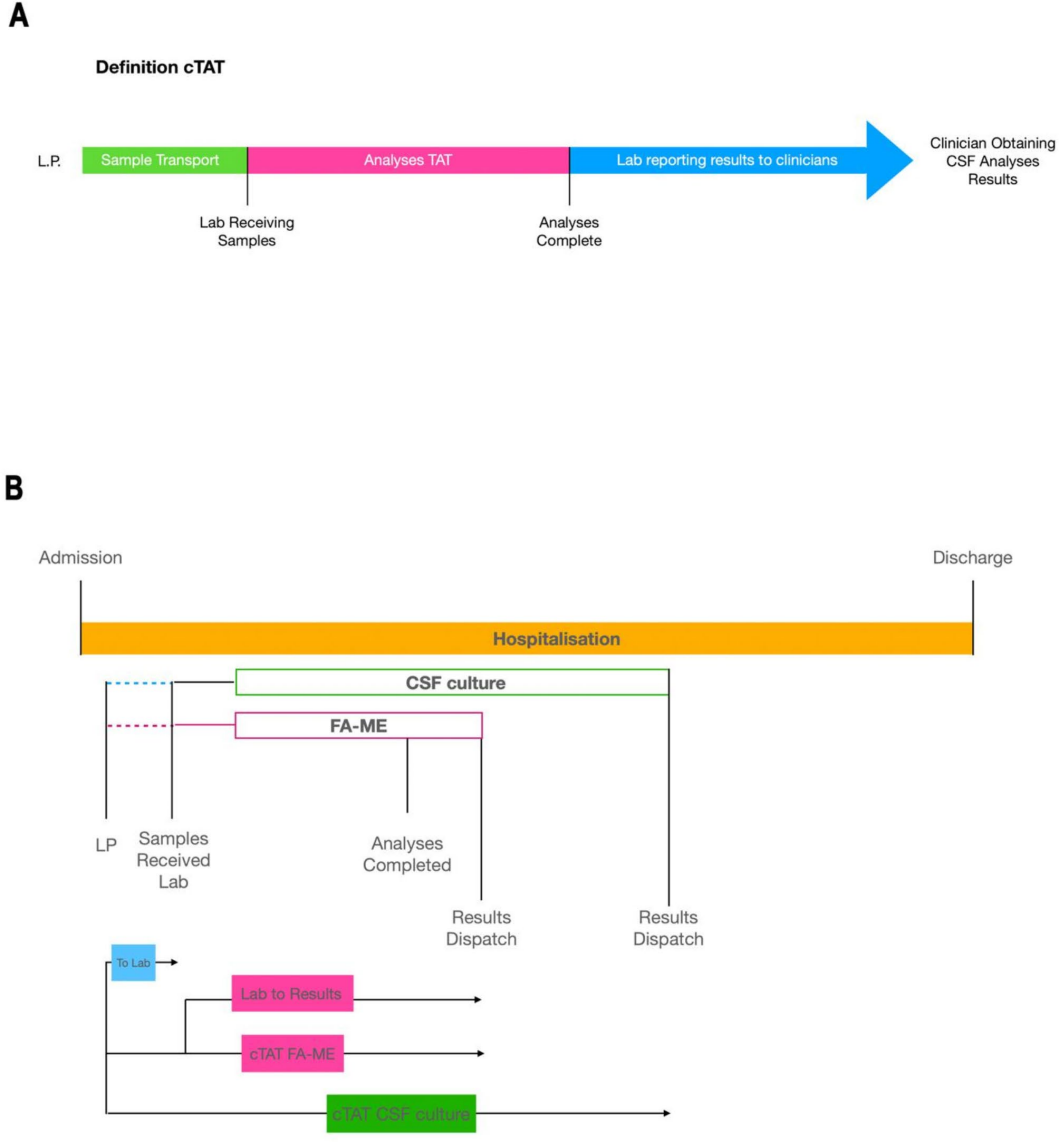
**A** and **B**. Schematic illustration of clinical turnaround time (cTAT); and cTAT illustrated as part of the total patient management time. cTAT = Clinical turnaround time. LP = Lumbar puncture. TAT = Turnaround time. CSF = Cerebrospinal fluid. FA-ME = FilmArray ME Panel

Previous studies have presented different definitions for turnaround time. Some authors have defined it as the time spent for sample analysis by the FA-ME instrument; others as time from LP to FA-ME results, without specifying whether the reporting of results to clinicians was included; or as laboratory time, i.e. from reception of sample to results being registered in electronic records [21, 31, 32]. In this study we defined clinical turnaround time (cTAT) as the time from LP to the time of clinicians receiving CSF analysis results (Fig. 2). cTAT of bacterial culture is presented in days and of FA-ME in hours. This is because patient records contain the exact time of reporting of FA-ME results to the hospitals, but for culture only the date.

### Ethical considerations

Ethical approvals for the PI-POC trial and all its sub-studies were received by the Institutional Ethical Review Board of Mbarara University of Science and Technology in Uganda (ref. 22/05–18) and the Regional Ethical Review Board in Stockholm, Sweden (ref. 2018/1676-31/1). Approval for the trial was granted by the Uganda National Council for Science and Technology (ref. HS 2508) in full compliance with the Declaration of Helsinki’s ethical principles. Spoken and written study information to, and informed consent from, guardians of potential participants were provided in local language. Patients received clinically indicated care or treatment, irrespective of study participation. As per requests from the local ethical review board, families of study participants were compensated with UGX 10,000, a daily meal during hospitalization, and a bar of soap.

Since no methods for microbiology (e.g. pathogen-specific PCR) other than bacterial culture and FA-ME were available in the setting, the study team considered it unethical to include a control group of participants with suspected CNS infection for which FA-ME analyses would then not be performed.

FA-ME and its reagents were purchased by the study team. FA-ME manufacturers had neither a supporting role for the study nor did they sponsor, provide gifts, or get involved in the design, conduct or writing of this manuscript.

## Results

**Fig. 3:**
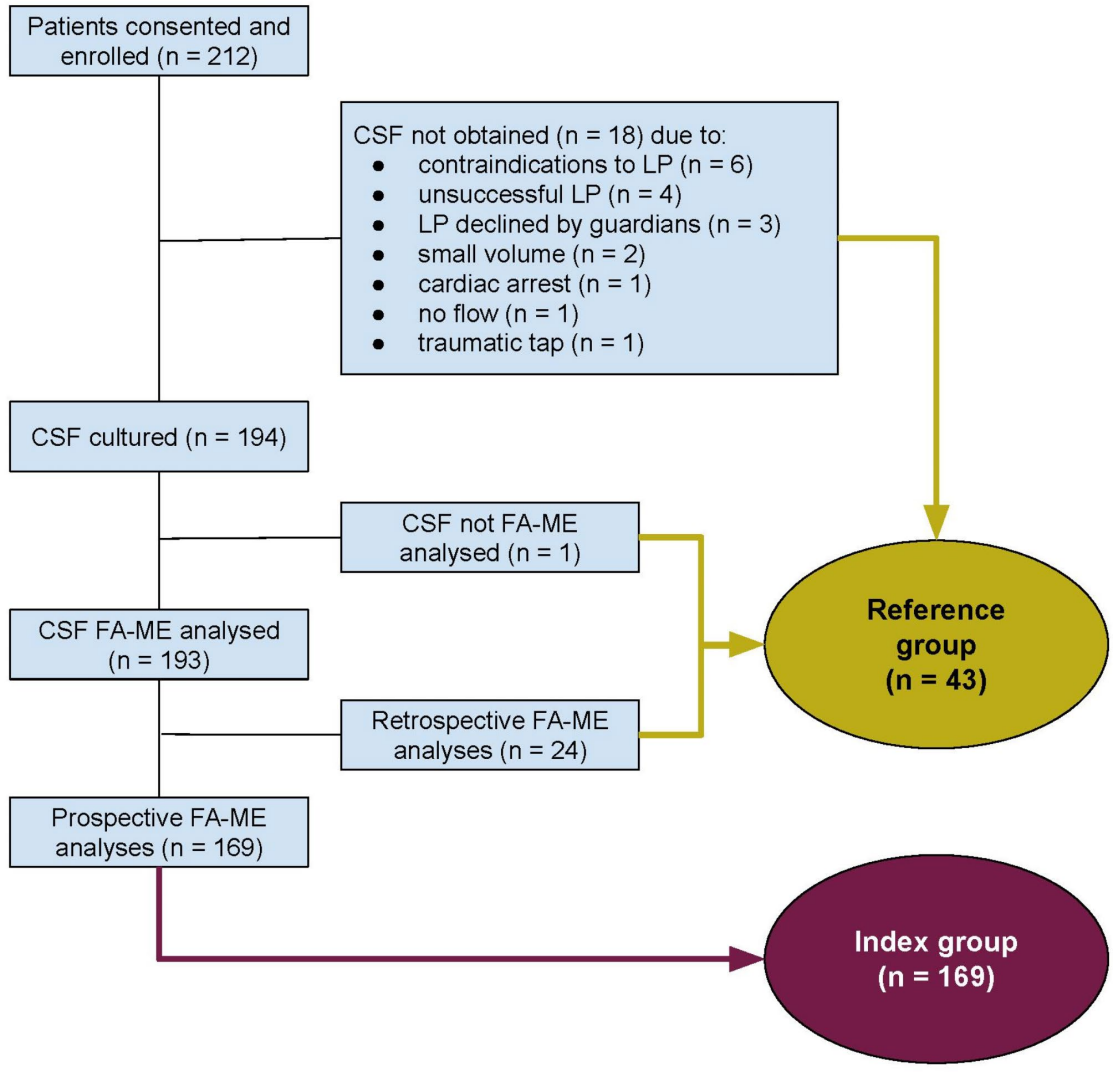
Flowchart of study participants. CSF = cerebrospinal fluid. FA-ME = FilmArray ME Panel. LP = Lumbar puncture

A total of 212 patients (89 from MRRH, 123 from HICH) were enrolled. LP was successful for 194 (92%) patients, allowing for bacterial culture for all collected CSF samples, and FA-ME analyses for 193 samples (Fig. 3). Median age for the 194 patients was 10 months (IQR 1–50) with 35% aged < 3 months, and 37% female. Microbiology results for these patients are presented in Table 1 and Appendix 2.

FA-ME analyses were prospectively conducted as part of the diagnostic work up for 169/193 (88%) patients, labelled as the ‘Index group’ (Fig. 3), for which cTAT was calculated.

In addition, a ‘Reference group’ (*n* = 43) emerged, comprising patients from whom CSF had been either not collected, or had undergone FA-ME analysis at a later stage, where results could not have influenced patient care.

### Clinical turnaround time for CSF microbiology

**Fig. 4:**
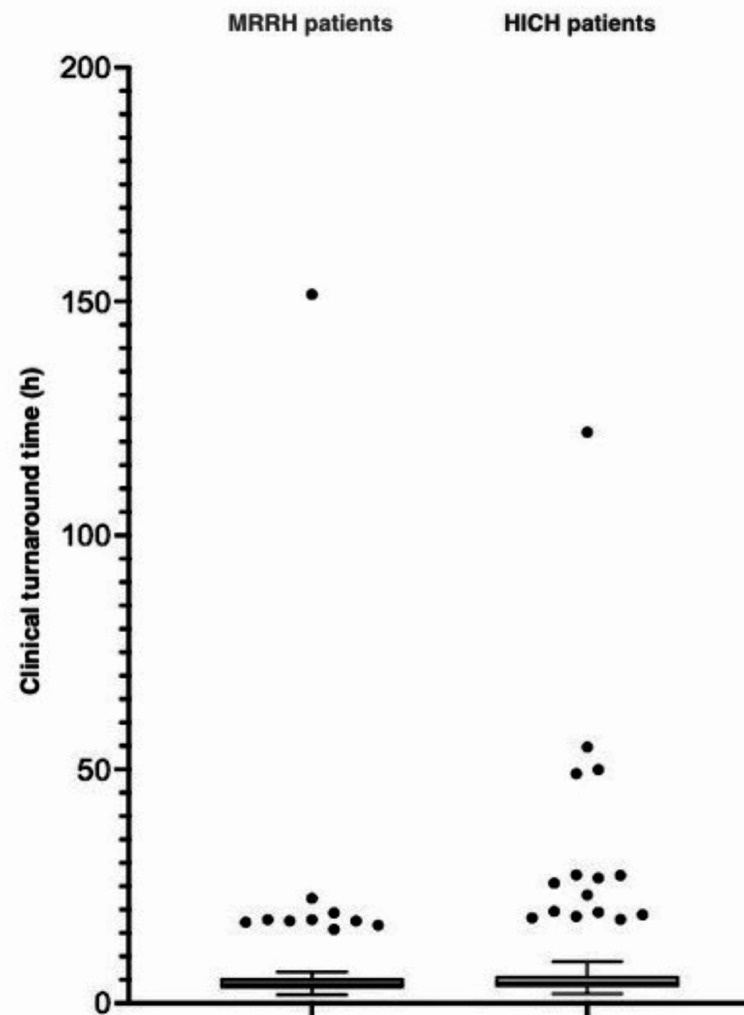
Clinical turnaround time (cTAT) of FA-ME analyses (hours),* broken down between hospitals.* MRRH = Mbarara Regional Referral Hospital. HICH = Holy Innocents Children’s Hospital

Median cTAT for FA-ME analyses was 4.2 h (IQR 3.3–5.5 h), with no significant difference seen between patients from MRRH (*n* = 67) and those from HICH (*n* = 102): 3.9 h vs. 4.3 h respectively (*P* = 0.15) (Fig. 4).

**Fig. 5:**
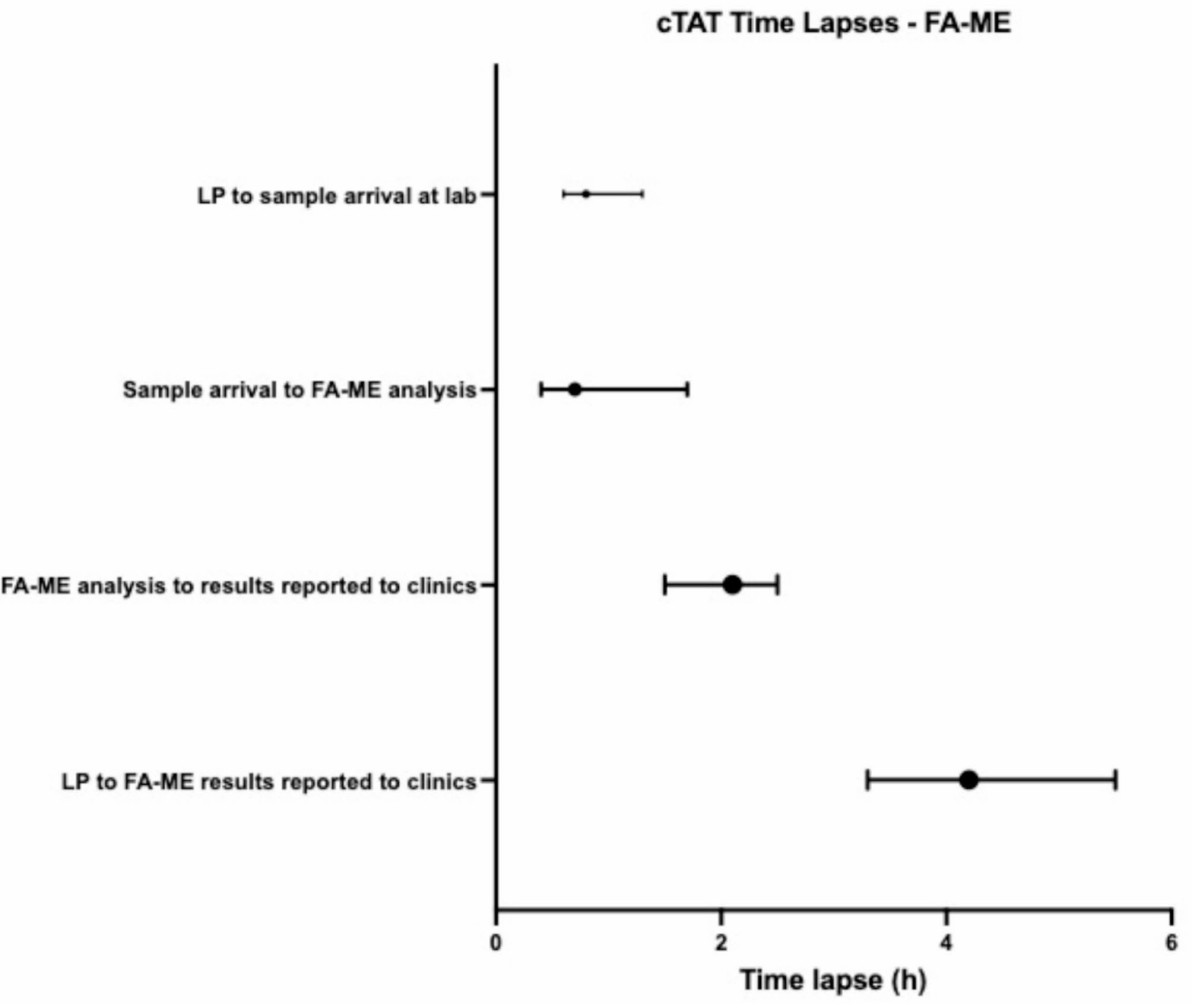
Breakdown of duration of each step (hours) of the clinical turnaround time (cTAT) of the FilmArray ME panel,* presented with median and IQR.* LP = Lumbar puncture. FA-ME = FilmArray ME Panel

The most time-consuming procedure was the laboratory reporting of FA-ME results to the hospitals, consuming a median of 2.1 h (IQR 1.5–2.5 h) (Fig. 5).

For CSF culture, median cTAT was 2 days (IQR 2–2 d) for all 169 patients together, as well as for each hospital individually.

### CSF analyses, microorganism detection, and pre-treatment with antibiotics

FA-ME detected bacteria in 24 (12%), and viruses in 23 (12%) of the analysed CSF samples, with a total of 40 (21%) samples showing sign of microorganisms (Table 1). In contrast, CSF culture yielded three (1.5%) bacteria-positive samples, which were also detected by FA-ME.

Human herpesvirus 6 (HHV-6) was the most common microorganism, detected in 19 (9.8%) samples, and *S. pneumoniae* the most common bacteria, detected in 11 (5.7%) samples. Of the FA-ME positive samples, 33 (83%) had one detected microorganism, whereas in seven (18%) samples, two microorganisms were detected.

Pre-treatment with antibiotics was reported in 168/194 (87%) patients < 7 days prior to LP, discontinued within 4–7 days from LP in two patients, and within < 4 days in three patients. Fifteen (7.7%) patients had no antibiotics exposure before LP, and antibiotics status was unknown for 11 (5.7%) patients, although not initiated at the hospital before LP.

Of the 168 pre-treated patients, 47 received antibiotics before admission. The remaining 121 were treated empirically after admission with Ceftriaxone alone or combined with Benzylpenicillin/Ampicillin, or Ampicillin with Gentamycin, before LP. There is no reliable data on antibiotic regimens for the 47 community-treated patients.

Of the 24 FA-ME bacteria-positive patients, 22 (92%) had received antibiotics within < 4 days of LP, one patient had not received any, and the status was unknown in one patient. Of the culture-positive patients, one had received antibiotics before LP, one had not, and the status was unknown for the third patient.

Of the 170 bacteria-negative patients: 127 (75%) had received antibiotics before LP and 14 (8.2%) had not; three (1.8%) had received antibiotics < 4 days before LP; one (0.6%) had received 4–7 days before LP; and for nine (5.3%) patients it was unknown.

*Listeria monocytogenes*, Herpes simplex virus 1 or 2, Human parechovirus, Varicella zoster virus, and *Cryptococcus neoformans/gatii* were not identified in any of the samples, even though detectable by FA-ME.

CSF White Blood Cell (WBC) count was done for 22 of the bacteria-positive samples, with a median count of 163 (IQR 0–1,038)/μL. Seven (32%) of these, including one FA-ME positive/culture-negative case of *N. meningitidis*, had neither non-clear CSF visual appearance nor pleocytosis (defined as WBC count ≥ 5/μL). Of the samples with no microorganism detection, pleocytosis was found in 17 (11%). An overview of CSF findings with patient outcomes is presented in Appendix 2.

**Table 1:** Microbiology results from Film Array ME panel and CSF culture

		FA-ME	Culture
		*n*	
**Strictly bacterial**	*Escherichia coli*	1	
	*Neisseria meningitidis*	4	1
	*Streptococcus agalactiae*	1	
	*Streptococcus pneumoniae*	8	1
	*Haemophilus influenzae*	3	1
**Strictly viral**	HHV-6*	12	
	*Cytomegalovirus*	2	
	*Enterovirus*	2	
**Mixed viral/bacterial**	*HHV-6 + E. coli*	1	
	*HHV-6 + S. pneumoniae*	3	
	*HHV-6 + S. agalactiae*	2	
	*HHV-6 + H. influenzae*	1	
Negative		153	191
Not analysed		1	-
Total		193	194

*Human herpesvirus 6

### Overall patient outcome

Of all 212 children with suspected CNS infection, 32 (15%) died, 142 (67%) were cured, and 38 (18%) had other or unknown outcome. The latter category included self-discharge against advice; patients lost during transfer between the two hospitals; transfer to hospitals outside the district; and one case of unconfirmed death where the family self-discharged before arrival of hospital personnel.

There was a non-significant higher case fatality rate for those with any microorganism detected in CSF (18%), compared to those without (13%) (*P* = 0.76).

### FA-ME influence on patient outcome

Case fatality rate for the Index group was 24/169 (14%), as compared to 8/43 (19%) for the Reference group, although the difference was not statistically significant (*P* = 0.20).

Similarly, for the Index group, there was no significant relationship between positive and negative FA-ME results, and for patient outcome (Table 2).

**Table 2:** Outcome of CSF positive vs. negative patients of the Index group

	Cured, *N* (%)	Death, *N* (%)	Unknown outcome, *N* (%)	Total, *N*
CSF positives	20 (61)	6 (18)	7 (21)	33
CSF negatives	98 (72)	18 (13)	20 (15)	136
Total	118	24	27	169

*P* = 0.44

### FA-ME influence on duration of antibiotic therapy

Of the Index group survivors, data on antibiotics duration was available for 104 patients. For the 96/104 patients for whom clinicians had received bacteria-negative (viral or all-negative) FA-ME results, antibiotics were discontinued after a median of six days (IQR 4–10). At one, two, three, and seven days after receipt of such a FA-ME result, 96%, 86%, 76%, and 36% of such patients remained on antibiotics, respectively.

For the 8/104 bacteria-positive patients, antibiotics were discontinued after a median of 13 days (IQR 9–13) from receipt of FA-ME results.

The difference between the two groups was statistically significant (*P* = 0.03).

### FA-ME influence on duration of hospitalization

There was no difference seen in median duration of hospitalization between the Index group and the Reference group: 8 days (IQR 5–14) vs. 9 days (IQR 4–15) (*P* = 0.89).

Comparing the 136 FA-ME negative to the 33 FA-ME positive patients of the Index group, a negative FA-ME result was associated with a significant reduction in median duration of hospitalization: 7 days (IQR 5–12) vs. 12 days (IQR 7.5–16) (*P* = 0.0007).

## Discussion

In this mainly prospective study conducted in Uganda where most enrolled children had been empirically treated with antibiotics prior to LP, we found the FilmArray® Meningitis/Encephalitis panel to provide clinicians with an increased bacterial yield in CSF microbiology and the enabling of viral detection, both at a shorter cTAT than what is typically achieved with CSF culture. Furthermore, FA-ME negative patients were hospitalized for five fewer days than FA-ME positive patients, and after receiving results, clinicians discontinued antibiotics more than twice as fast for bacteria-negative than bacteria-positive patients, although discontinuation took several days. Even though a possible explanation for shorter hospitalization and antibiotic exposure could be that FA-ME negative patients would be less severely ill than their positive counterparts, fatality rates were not significantly different between groups.

Neither could a significant difference in mortality be found between patients with suspected CNS infection for whom diagnosis had been supported by FA-ME or not.

Previous studies have presented different definitions for turnaround time [21, 31, 32]. We argue that defining cTAT as time from LP to time of clinicians receiving results of sample analyses better correspond to the clinical *utility* of the analysis method and is in line with the scope of the study.

In line with information from the FA-ME manufacturer, laboratory turnaround time of FA-ME analyses was drastically shorter than that of bacterial culture in this study. Also, with the logistical process implemented for this study, we found that the overall cTAT – from LP to clinicians receiving FA-ME results – was equally shorter than that of culture. As there was no difference in cTAT observed between the two hospitals (MRRH being adjacent to the laboratory and HICH at a 2.5 km distance), it would seem that a FA-ME instrument need not necessarily be installed at each point of patient care for its clinical utilities to be gained, if a well-established supply chain for sample and results delivery is in place. In this study, potential delays in results delivery to the more distant hospital were mitigated by electronic reporting. The cTAT of even the most rapid diagnostic instrument is highly dependent on the logistical processes of the health system in which it operates [10, 33]. As about half of the FA-ME cTAT was spent on reporting results to clinicians, further reductions of cTAT could probably be accomplished if the delay is addressed.

The ability of FA-ME to detect bacteria in eight times as many patients than through culture, as found in this study, suggests a superior sensitivity of its molecular methodology in settings where empiric antibiotic treatment before LP is routine. This is echoed by a retrospective Nigerian study where FA-ME detected bacteria in 30.5% of CSF samples, as compared to 8% through culture [34]. Furthermore, while no alternate method for viral detection was available during the study period, FA-ME detected viruses in 23 CSF samples that would otherwise have been undetected. However, these detections have not been confirmed in this study, and some reports shed doubt on the accuracy and significance of some FA-ME results. Frequent HHV-6 and Cytomegalovirus detection by FA-ME is reported by several authors, who in most cases considered them non-pathogenic due to their latency capacity and chromosomal integration (HHV-6), and non-significance in immunocompetent patients (Cytomegalovirus) [17, 20, 31, 34–36]. False positive streptococcal and false negative Herpes simplex virus 1/2 and *C. neoformans/gatii* detections have been previously reported [13, 18, 22, 37]. Such false results could have potential implications for the patients of this study, where streptococci were the most frequently detected bacteria, while no Herpes simplex virus or cryptococci were detected. Also, in a report from Spain, FA-ME failed to detect *N. meningitidis* in a patient despite CSF characteristics strongly indicative of bacterial meningitis [38]. In another recent study on a HIV-positive cohort in Mbarara, Uganda, two cases of *S. pneumoniae* detection by FA-ME were considered as sample contaminations [22]. As nearly four-tenths of our FA-ME bacteria-positive cases had normal visual appearance, CSF WBC count, and culture of CSF (Appendix 2), contamination or false positives cannot completely be ruled out.

Investigators of a study in Ethiopia (including children and adults) found a WBC count cut-off value of ≥75/μL_CSF_ for all their patients with bacterial CNS infection [23]. This could not be reproduced in our study, where 32% of bacteria-positive CSF samples either lacked pleocytosis or had a WBC count below the proposed cut-off. Also, the CSF of one of our patients had neither pleocytosis nor abnormal visual appearance, despite FA-ME detecting *N. meningitidis*. For another study patient, the FA-ME result was negative, although its CSF showed pleocytosis (WBC count 120/μL) (Appendix 2). Even though the absence of pleocytosis in children (and especially neonates) with confirmed CNS infection has been reported [16, 39, 40], our findings could also represent sample contamination, or false FA-ME results.

In a study conducted 2009–2012, on the same target population and setting as this study, cerebral malaria was the most frequent cause of CNS infection, and, non-typhoidal *Salmonella* (which is currently not targeted by FA-ME) to be the second most frequent bacterial cause [41]. Similarly in Nigeria, three instances of *Salmonella* species were only detectable through confirmatory testing by an alternative PCR panel [34]. Another microorganism not targeted by FA-ME is *Mycobacterium tuberculosis*, despite it being a major cause of childhood CNS infection in tuberculosis endemic areas such as Uganda [6, 20]. Hence, if they are to use FA-ME as a stand-alone CSF microbiology method, clinicians in settings such as those in this study must keep in mind that it does not currently encompass the full spectra of childhood CNS infection aetiology everywhere. However, as the manufacturer was able to add the SARS-CoV-2 virus to the targets of its pre-existing FilmArray® Respiratory panel in less than one year, an extension of the Meningitis/Encephalitis panel with microorganisms, such as non-typhoidal *Salmonella*, would better correspond to local epidemiology and improve its microbial yield and clinical utility [20, 42].

With respect to the feasibility of its use in low-resource settings, the FA-ME instrument itself costs approximately USD 35,000, and the test cartridges for each analysis an additional USD 150 per test. Even though reports from Greece and the United States have described an overall cost-effectiveness regarding the use of FA-ME, one has to contrast the annual health expenditure per capita of Greece (USD 1,600) and the United States (USD 10,600) to that in Uganda (USD 43) and low-income countries in general (USD 35) [1, 14, 43]. Although with a functioning supply chain of samples and results, the purchase cost of the instrument could be shared by several collaborative caregivers in close vicinity, the high cost for every single analysis would not be diminished by such a partnership.

Additional disadvantages of FA-ME observed during the study were: short expiry of FA-ME test cartridges; logistical challenges in delivery (of the instrument and cartridges) from, and maintenance and service by, the nearest supplier in a neighbouring country; the need for a connected computer to run the manufacturer’s software needed for its operation; and its need for a stable power grid, which has been described in another report [22]. Also, FA-ME does not provide antimicrobial susceptibility testing of detected microorganisms. However, considering its short turnaround time, susceptibility testing of only bacteria-positive samples could be initiated in the laboratory after FA-ME results, without substantial time loss. This could potentially result in a higher yield than blind susceptibility testing, while preserving limited resources.

### Strengths and limitations

This study focused on children with suspected CNS infection and the clinical utility of FA-ME for diagnosis. It did not account for other potential causes of illness, like sepsis or cerebral malaria, as the inclusion criteria were pre-defined for CNS infection. Additionally, confirmatory testing with alternative molecular methods such as GeneXpert and Rapid Diagnostic Tests such as Cryptococcal Antigen test (CrAg) were not routinely performed, inhibiting the possibility to analyse the diagnostic accuracy of FA-ME, although the primary focus was on clinical utility. The study also lacked a formal control group where FA-ME was not used. While the inclusion of such a group was considered during study planning, it was deemed unethical due to limitations in CSF culture yield caused by routine use of empiric antibiotics prior to lumbar puncture.

Although, as previously described, a small reference group was made possible, its emergence was unplanned for, and thus it was not possible to ensure the matching of its participants’ characteristics to those of the Index group. Seven participants of the Reference group showed signs (contraindication to LP and cardiac arrest) of severe clinical condition. This could have caused selection bias, affecting the study outcome measures to the disbenefit of the Reference group. Another factor limiting generalizability of the study is its potential elevation of the baseline supply chain structure between the two hospitals and the laboratory. Also, the choice of the laboratory used to conduct analyses influences the generalizability of study findings. By virtue of its international accreditation, Epicentre laboratory holds to standards of quality that cannot be matched by most laboratories in Uganda, or even in the region. At the same time, it would have been difficult to conduct the study without such logistics in place. With the mainly prospective field design of this study and its independence from the instrument manufacturer, we believe our findings to be credible and representative of the clinical performance of FA-ME in Sub-Saharan settings with limited health resources.

## Conclusions

In conclusion, our study suggests that multiplex molecular methods like FA-ME can offer substantial clinical utility to low-resource healthcare systems when integrated with a well-functioning supply chain. This is made possible by enhancing microbial yield at a reduced clinical turnaround time and shortened hospitalization and antibiotic exposure for patients without CSF pathology. However, while FA-ME provides a broader microorganism targeting spectrum compared to traditional methods in the study setting, it may not fully represent local childhood CNS infection epidemiology. Expanding the FA-ME panel or considering region-specific panels could improve its utility. The significant obstacle to its routine use in low-resource healthcare systems is its high cost. Subsidies or cost-shifting strategies are essential for making FA-ME or similar instruments feasible in low-resource settings [22].

Additionally, a substantial number of children who underwent LP due to suspected CNS infection showed no signs of infection in their CSF. This underscores the dire need for less invasive methods to screen patients eligible for LP. Such methods would not only reduce the discomfort experienced by children but also better household with the limited resources.

## Electronic supplementary material

Below is the link to the electronic supplementary material.

Appendix 1

Appendix 2

## Data Availability

The datasets used and/or analysed during the current study are available from the corresponding author on reasonable request.

## Abbreviations

CNS: Central nervous system
CSF: Cerebrospinal fluid
cTAT: Clinical turnaround time
FA-ME: FilmArray Meningitis Encephalitis Panel
HHV-6: Human herpesvirus 6
HICH: Holy Innocents Children’s Hospital
IQR: Interquartile range
LP: Lumbar puncture
LRS: Low-resource settings
MRRH: Mbarara Regional Referral Hospital
PCR: Polymerase chain reaction
PI-POC: Paediatric Infections Point-of-Care Trial
WBC: White blood cell

## References

[1] Posnakoglou L, Siahanidou T, Syriopoulou V, Michos A. Impact of cerebrospinal fluid syndromic testing in the management of children with suspected central nervous system infection. Eur J Clin Microbiol Infect Dis. 2020;39(12):2379–86. 32683594 10.1007/s10096-020-03986-6

[2] Singhi P. Central nervous system infections in children: an ongoing challenge! Indian J Pediatr. 2019;86(1):49–51. 30132192 10.1007/s12098-018-2745-6

[3] Polage CR, Cohen SH. State-of-the-art microbiologic testing for community-acquired meningitis and encephalitis. J Clin Microbiol. 2016;54(5):1197–202. 26888896 10.1128/JCM.00289-16 PMC4844713

[4] Sáez-Llorens X, McCracken GH. Bacterial meningitis in children. Lancet. 2003;361(9375):2139–48. 12826449 10.1016/S0140-6736(03)13693-8

[5] Nauclér P, Huttner A, Van Werkhoven CH, Singer M, Tattevin P, Einav S, et al. Impact of time to antibiotic therapy on clinical outcome in patients with bacterial infections in the emergency department: implications for antimicrobial stewardship. Clin Microbiol Infect. 2021;27(2):175–81. 32120032 10.1016/j.cmi.2020.02.032

[6] Robertson FC, Lepard JR, Mekary RA, Davis MC, Yunusa I, Gormley WB et al. Epidemiology of central nervous system infectious diseases: a meta-analysis and systematic review with implications for neurosurgeons worldwide. J Neurosurg. 2019;130(4):1107–26. 10.3171/2017.10.JNS17359 29905514

[7] Institute for Health Metrics and Evaluation: Global Burden of Disease Collaborative Network. Global burden of disease study 2019 (GBD 2019) reference life table. Seattle, United States of America: Institute for Health Metrics and Evaluation (IHME); 2021. [Available from: ghdx.healthdata.org/.

[8] Laxminarayan R, Duse A, Wattal C, Zaidi AKM, Wertheim HFL, Sumpradit N, et al. Antibiotic resistance—the need for global solutions. Lancet Infect Dis. 2013;13(12):1057–98. 24252483 10.1016/S1473-3099(13)70318-9

[9] Urdea M, Penny LA, Olmsted SS, Giovanni MY, Kaspar P, Shepherd A, et al. Requirements for high impact diagnostics in the developing world. Nature. 2006;444(Suppl 1):73–9. 17159896 10.1038/nature05448

[10] Rasti R, Nanjebe D, Karlstrom J, Muchunguzi C, Mwanga-Amumpaire J, Gantelius J, et al. Health care workers’ perceptions of point-of-care testing in a low-income country-a qualitative study in Southwestern Uganda. PLoS ONE. 2017;12(7):e0182005. 28750083 10.1371/journal.pone.0182005 PMC5547696

[11] Petti CA, Polage CR, Quinn TC, Ronald AR, Sande MA. Laboratory medicine in Africa: a barrier to effective health care. Clin Infect Dis. 2006;42(3):377–82. 16392084 10.1086/499363

[12] Davis LE. Acute bacterial meningitis. Continuum (Minneap Minn). 2018;24(5):1264–83. 30273239 10.1212/CON.0000000000000660

[13] Lee SH, Chen S-Y, Chien J-Y, Lee T-F, Chen J-M, Hsueh P-R. Usefulness of the filmarray meningitis/encephalitis (M/E) panel for the diagnosis of infectious meningitis and encephalitis in Taiwan. J Microbiol Immunol Infect. 2019;52(5):760–8. 31085115 10.1016/j.jmii.2019.04.005

[14] Soucek DK, Dumkow LE, Vanlangen KM, Jameson AP. Cost justification of the biofire filmarray meningitis/encephalitis panel versus standard of care for diagnosing meningitis in a community hospital. J Pharm Pract. 2019;32(1):36–40. 29092659 10.1177/0897190017737697

[15] World Health Organization. Defeating meningitis by 2030: a global road map. 2021 [Available from: https://www.who.int/publications/i/item/9789240026407

[16] Naccache SN, Lustestica M, Fahit M, Mestas J, Dien Bard J. One year in the life of a rapid syndromic panel for meningitis/encephalitis: a pediatric tertiary care facility’s experience. J Clin Microbiol. 2018;56(5). 10.1128/JCM.01940-17 PMC5925728 29540454

[17] Leber AL, Everhart K, Balada-Llasat JM, Cullison J, Daly J, Holt S, et al. Multicenter evaluation of biofire filmarray meningitis/encephalitis panel for detection of bacteria, viruses, and yeast in cerebrospinal fluid specimens. J Clin Microbiol. 2016;54(9):2251–61. 27335149 10.1128/JCM.00730-16 PMC5005480

[18] Tansarli GS, Chapin KC. Diagnostic test accuracy of the BioFire® FilmArray® meningitis/encephalitis panel: a systematic review and meta-analysis. Clin Microbiol Infect. 2020;26(3):281–90. 31760115 10.1016/j.cmi.2019.11.016

[19] bioMérieux. FilmArray Meningitis/Encephalitis (ME) Panel 2017 [Available from: http://www.biomerieux-diagnostics.com/filmarray-meningitis-encephalitis-me-panel

[20] Vetter P, Schibler M, Herrmann JL, Boutolleau D. Diagnostic challenges of central nervous system infection: extensive multiplex panels versus stepwise guided approach. Clin Microbiol Infect. 2020;26(6):706–12. 31899336 10.1016/j.cmi.2019.12.013

[21] Radmard S, Reid S, Ciryam P, Boubour A, Ho N, Zucker J et al. Clinical utilization of the filmarray meningitis/encephalitis (ME) multiplex polymerase chain reaction (PCR) assay. Front Neurol. 2019;10(281). 10.3389/fneur.2019.00281 PMC6443843 30972012

[22] Bridge S, Hullsiek KH, Nerima C, Evans EE, Nuwagira E, Stadelman AM, et al. Evaluation of the BioFire® FilmArray® meningitis/encephalitis panel in an adult and pediatric Ugandan population. J Med Mycol. 2021;31(3):101170. 10.1016/j.mycmed.2021.101170 PMC9983612 34246087

[23] Bårnes GK, Gudina EK, Berhane M, Abdissa A, Tesfaw G, Abebe G, et al. New molecular tools for meningitis diagnostics in Ethiopia - a necessary step towards improving antimicrobial prescription. BMC Infect Dis. 2018;18(1):684. 30572843 10.1186/s12879-018-3589-4 PMC6302510

[24] Gaudenzi G, Kumbakumba E, Rasti R, Nanjebe D, Réu P, Nyehangane D, et al. Point-of-care approaches for meningitis diagnosis in a low-resource setting (Southwestern Uganda): observational cohort study protocol of the PI-POC trial. JMIR Res Protocols. 2020;9(11):e21430. 10.2196/21430 PMC7690656 33146628

[25] Uganda Bureau of Statistics. End of Month Population Projections 2015 to 2040 2020 [Available from: https://www.ubos.org/explore-statistics/20/

[26] Schiess N, Groce NE, Dua T. The impact and burden of neurological sequelae following bacterial meningitis: a narrative review. Microorganisms. 2021;9(5):900. 33922381 10.3390/microorganisms9050900 PMC8145552

[27] United Nations Inter-agency Group for Child Mortality Estimation (UN IGME). Levels & Trends in Child Mortality: Report 2020, Estimates developed by the United Nations Inter-agency Group for Child Mortality Estimation 2020 [Available from: www.childmortality.org

[28] MSF Epicentre. Website of Epicentre Mbarara Research Center 2021 [Available from: https://epicentre.msf.org/en/epicentre/research-center-uganda

[29] Harris PA, Taylor R, Minor BL, Elliott V, Fernandez M, O’Neal L, et al. The REDCap consortium: building an international community of software platform partners. J Biomed Inf. 2019;95:103208. 10.1016/j.jbi.2019.103208 PMC7254481 31078660

[30] Harris PA, Taylor R, Thielke R, Payne J, Gonzalez N, Conde JG. Research electronic data capture (REDCap)--a metadata-driven methodology and workflow process for providing translational research informatics support. J Biomed Inf. 2009;42(2):377–81. 10.1016/j.jbi.2008.08.010 PMC2700030 18929686

[31] Chong BSW, Kennedy KJ. Comparison of a commercial real-time PCR panel to routine laboratory methods for the diagnosis of meningitis-encephalitis. Pathology. 2021;53(5):635–8. 10.1016/j.pathol.2020.09.029 33472744

[32] Messacar K, Breazeale G, Robinson CC, Dominguez SR. Potential clinical impact of the film array meningitis encephalitis panel in children with suspected central nervous system infections. Diagn Microbiol Infect Dis. 2016;86(1):118–20. 27342782 10.1016/j.diagmicrobio.2016.05.020 PMC4993637

[33] Katoba J, Kuupiel D, Mashamba-Thompson TP. Toward improving accessibility of point-of-care diagnostic services for maternal and child health in low- and middle-income countries. Point Care. 2019;18(1):17–25. 30886544 10.1097/POC.0000000000000180 PMC6407818

[34] Obaro S, Hassan-Hanga F, Medugu N, Olaosebikan R, Olanipekun G, Jibir B et al. Comparison of bacterial culture with BioFire® FilmArray® multiplex PCR screening of archived cerebrospinal fluid specimens from children with suspected bacterial meningitis in Nigeria. BMC Infect Dis. 2023;23(1). 10.1186/s12879-023-08645-7 PMC10544496 37784010

[35] Säll O, Thulin Hedberg S, Neander M, Tiwari S, Dornon L, Bom R, et al. Etiology of central nervous system infections in a rural area of Nepal using molecular approaches. Am J Trop Med Hyg. 2019;101(1):253–9. 31162021 10.4269/ajtmh.18-0434 PMC6609203

[36] Eichinger A, Hagen A, Meyer-Bühn M, Huebner J. Clinical benefits of introducing real-time multiplex PCR for cerebrospinal fluid as routine diagnostic at a tertiary care pediatric center. Infection. 2019;47(1):51–8. 30187216 10.1007/s15010-018-1212-7

[37] Lindström J, Elfving K, Lindh M, Westin J, Studahl M. Assessment of the filmarray ME panel in 4199 consecutively tested cerebrospinal fluid samples. Clin Microbiol Infect. 2021;28(1):79–84. 10.1016/j.cmi.2021.05.017 34015534

[38] González-Donapetry P, García-Rodríguez J, Cendejas-Bueno E. A case of a FilmArray® ME false negative in meningococcal meningitis. J Infect. 2019;79(3):277–87. 10.1016/j.jinf.2019.05.002 31102602

[39] Yun KW, Choi EH, Cheon DS, Lee J, Choi CW, Hwang H, et al. Enteroviral meningitis without pleocytosis in children. Arch Dis Child. 2012;97(10):874–8. 22814522 10.1136/archdischild-2012-301884

[40] Garges HP. Neonatal meningitis: what is the correlation among cerebrospinal fluid cultures, blood cultures, and cerebrospinal fluid parameters?? Pediatrics. 2006;117(4):1094–100. 16585303 10.1542/peds.2005-1132

[41] Page AL, Boum Ii Y, Kemigisha E, Salez N, Nanjebe D, Langendorf C, et al. Aetiology and outcomes of suspected infections of the central nervous system in children in Mbarara, Uganda. Sci Rep. 2017;7(1):2728. 28578421 10.1038/s41598-017-02741-w PMC5457409

[42] First of 3 diagnostic tests for SARS-CoV-. 2 coronavirus available from bioMérieux [press release]. https://www.biomerieux.com/en/novel-coronavirus-covid-19, 11 March.

[43] World Health Organization. Global Health Expenditure database 2018 [Available from: https://apps.who.int/nha/database

