# Supplementary material for: Clinical utility of the FilmArray® meningitis/encephalitis panel in children with suspected central nervous system infection in a low-resource setting – a prospective study in Southwestern Uganda
Source: BMC Infect Dis. 2025 Mar 22;25:396. doi: 10.1186/s12879-025-10732-w (PMC11930002; doi:10.1186/s12879-025-10732-w)
Supplement: Supplementary file 1 — Appendix 1 [file 12879_2025_10732_MOESM1_ESM.docx]

# **Appendix 1.** Inclusion criteria

Copied from: Gaudenzi, G., et al., Point-of-Care Approaches for Meningitis Diagnosis in a Low-Resource Setting (Southwestern Uganda): Observational Cohort Study Protocol of the “PI-POC” Trial. JMIR Research Protocols, 2020. 9(11): p. e21430.

Children are suspected to have a CNS infection if they have fever or a history of fever in the past 48 hours (except for children younger than 9 months who may present with fever, normal body temperature, or hypothermia) and recent onset of any of the following at inclusion:

- Nontraumatic reduced level of consciousness (in preverbal children, this corresponds to Blantyre coma score <4 for those aged <9 months, and <5 for those older than 9 months (up to 12 years of age); in verbal children, this corresponds to Glasgow Coma Scale score <15);
- Prostration, hypotonia/hypertonia, unexplained irritability;
- Severe headache (severe enough to require hospitalization);
- Photophobia;
- Neck stiffness or bulging fontanel;
- Prolonged, partial, or multiple seizure(s);
- Focal neurological signs;
- In children older than 18 months: Kernig sign (flexion of the hip 90° with subsequent pain in legs extension) or Brudzinski sign (involuntary flexion of the knees and hips after passive flexion of the neck);
- Skin petechiae;
- Cheyne–Stokes breathing;

Cases are recruited from the emergency department and inpatient ward of HICH and MRRH, when clinical presentation instigates suspicion of CNS infection and when the aforesaid inclusion criteria have been met. However, due to the often-ambiguous presentation of CNS infection symptoms, attending medical officers may include suspected cases even if the stated criteria are not met. The inclusion criteria of those children will be clearly mentioned in the case report form, and inclusion must be validated by the principal investigator in Mbarara.
