# Supplementary material for: Clinical utility of the FilmArray® meningitis/encephalitis panel in children with suspected central nervous system infection in a low-resource setting – a prospective study in Southwestern Uganda
Source: BMC Infect Dis. 2025 Mar 22;25:396. doi: 10.1186/s12879-025-10732-w (PMC11930002; doi:10.1186/s12879-025-10732-w)
Supplement: Supplementary file 2 — Appendix 2 [file 12879_2025_10732_MOESM2_ESM.docx]

# **Appendix 2.** Summary of findings and outcomes in patients with detected microorganisms and those with elevated CSF WBC count but no detected microorganisms.

| **Patients with detected microorganisms in CSF** | | | | | | **Patients with elevated WBC count (≥5/μL) but no microorganisms in CSF** | | | | |
| --- | --- | --- | --- | --- | --- | --- | --- | --- | --- | --- |
| **Age (months)** | **Microorganisms** | **AB**  **before LP** | **Appearance** | **WBC count** | **Outcome** | **Age (months)** | **Appearance** | **WBC count** | **AB before LP** | **Outcome** |
| 146* | *S. pneumoniae* | Yes | Purulent | 750 | Cured | 3* | Clear | 40 | Yes | Cured |
| 2* | *S. pneumoniae* | Yes | Clear | 5 | Cured | 104* | Clear | 10 | No | Cured |
| 10* | *S. pneumoniae* | Yes | Clear | 0 | Cured | 17* | Clear | 30 | No | Death |
| 11* | *E. coli* + HHV-6 | Yes | Clear | 0 | Death | 42 | Clear | 9 | DC 7 d  Before LP | Cured |
| 5*a | *S. pneumoniae* | Yes | Turbid | 500 | Cured | 87 | Clear | 12 | Yes | Cured |
| 69a | *N. meningitidis* | No | Turbid | 220 | Cured | 37 | Clear | 5 | Yes | Cured |
| 6a,b | *H. influenzae* | Unknown | Turbid | 2420 | Other/u.k.b | 139 | Clear | 7 | Yes | Cured |
| 1 | HHV-6 | Unknown | Blood stained | Not done | Other/u.k. | 0 | Blood stained | 10 | Yes | Other/u.k. |
| 0 | *S. pneumoniae +* HHV-6 | Yes | Blood stained | Non applicable | Cured | 70 | Clear | 10 | Yes | Cured |
| 0 | *S. pneumoniae* | Yes | Turbid | 1050 | Death | 69 | Clear | 120 | Yes | Cured |
| 0 | *E. coli* | Yes | Turbid | 106 | Death | 9 | Clear | 9 | Yes | Other/u.k. |
| 0 | *S. agalactiae* | Yes | Xantochromic | 10 | Cured | 0 | Xantochromic | 5 | Yes | Cured |
| 1 | CMV | Yes | Blood stained | Non applicable | Cured | 47 | Clear | 6 | Yes | Cured |
| 5 | *S. agalactiae* + HHV-6 | Yes | Turbid | 1950 | Cured | 0 | Clear | 9 | Yes | Cured |
| 9 | HHV-6 | Yes | Blood stained | Non applicable | Cured | 0 | Clear | 16 | Yes | Cured |
| 4 | Enterovirus | Yes | Clear | 0 | Cured | 8 | Clear | 6 | Yes | Cured |
| 88 | HHV-6 | DC 4 d before LP | Clear | 0 | Cured | 0 | Blood stained | 20 | Yes | Death |
| 32 | HHV-6 | Yes | Clear | 0 | Cured |  |  |  |  |  |
| 4 | *S. pneumoniae* + HHV-6 | Yes | Clear | 0 | Other/u.k. |  |  |  |  |  |
| 4* | *N. meningitidis* | Yes | Clear | 0 | Cured |  |  |  |  |  |
| 12* | *H. influenzae* | Yes | Blood stained | 0 | Cured |  |  |  |  |  |
| 1 | *H. influenzae* + HHV-6 | Yes | Turbid | 280 | Other/u.k. |  |  |  |  |  |
| 6 | HHV-6 | Yes | Clear | 0 | Cured |  |  |  |  |  |
| 7 | HHV-6 | Yes | Blood stained | Not done | Cured |  |  |  |  |  |
| 6 | CMV | Yes | Clear | 0 | Death |  |  |  |  |  |
| 18 | *H. influenae* | Yes | Clear | 5 | Cured |  |  |  |  |  |
| 0 | HHV-6 | Yes | Clear | 0 | Other/u.k. |  |  |  |  |  |
| 32 | Enterovirus | Yes | Clear | 4 | Cured |  |  |  |  |  |
| 0 | *S. pneumoniae* | Yes | Blood stained | Not done | Cured |  |  |  |  |  |
| 32 | HHV-6 | Yes | Clear | 2 | Death |  |  |  |  |  |
| 1 | *N. meningitidis* | Yes | Turbid | 1000 | Other/u.k. |  |  |  |  |  |
| 0 | *S. pneumoniae* + HHV-6 | Yes | Clear | 0 | Cured |  |  |  |  |  |
| 1 | HHV-6 | Yes | Clear | 0 | Death |  |  |  |  |  |
| 80 | HHV-6 | Yes | Clear | 0 | Cured |  |  |  |  |  |
| 1 | HHV-6 | Yes | Clear | 0 | Cured |  |  |  |  |  |
| 6 | *S. pneumoniae* | Yes | Turbid | 4050 | Death |  |  |  |  |  |
| 3 | *S. pneumoniae* | Yes | Clear | 0 | Cured |  |  |  |  |  |
| 21 | *N. meningitidis* | Yes | Turbid | 2214 | Cured |  |  |  |  |  |
| 1 | *S. agalacticae* + HHV-6 | Yes | Turbid | 1140 | Cured |  |  |  |  |  |
| 0 | HHV-6 | Yes | Xantochromic | 1 | Other/u.k. |  |  |  |  |  |
| Cerebrospinal fluid findings in all cases with microorganisms detected, and cases without microorganism detection yet with elevated CSF cell count.  *Retrospectively analysed on FAME. aBacteria detected by both culture and FAME. bUnconfirmed report of fatal outcome.  CSF = cerebrospinal fluid. AB = antibiotics. DC = discontinued. u.k. = Unknown. LP = Lumbar puncture. WBC = white blood cell count (in CSF). HHV-6 = Human herpesvirus 6. CMV = Cytomegalovirus. | | | | | | | | | | |
